# Likes, Shares, and Symptoms: The Hidden Toll of Early Adolescents’ Social Media Use on Well-Being

**DOI:** 10.3390/ijerph22010131

**Published:** 2025-01-20

**Authors:** Lisanne Vonk, Polina Putrik, Thérèse van Amelsvoort, Elien Vanluydt, Philippe Delespaul, Mark Levels, Tim Huijts

**Affiliations:** 1 Research Centre for Education and the Labour Market (ROA), School of Business and Economics, Maastricht University, P.O. Box 616, 6200 MD Maastricht, The Netherlandsm.levels@maastrichtuniversity.nl (M.L.); t.huijts@maastrichtuniversity.nl (T.H.); 2 Department of Social Medicine, Care and Public Health Research Institute (CAPHRI), Faculty of Health Medicine and Life Sciences (FHML), Maastricht University, P.O. Box 616, 6200 MD Maastricht, The Netherlands; polina.putrik@maastrichtuniversity.nl; 3 Living Lab Public Health Mosa, Department of Knowledge & Innovation, Public Health Service South Limburg (GGD Zuid Limburg), P.O. Box 33, 6400 AA Heerlen, The Netherlands; 4 Department of Psychiatry & Neuropsychology, Mental Health and Neuroscience Institute, Maastricht University, P.O. Box 616, 6200 MD Maastricht, The Netherlands; t.vanamelsvoort@maastrichtuniversity.nl (T.v.A.); ph.delespaul@maastrichtuniversity.nl (P.D.); 5 Mondriaan Mental Health Centre, P.O. Box 4436, 6401 CX Maastricht, The Netherlands

**Keywords:** social media, primary school, mental health, physical health, well-being

## Abstract

Social media use has rapidly increased over the past decade, especially among young people. To obtain more insight into the potential negative associations with problematic social media use in Dutch early adolescents, we assessed its relation to self-reported well-being. We conducted a cross-sectional study with 585 students in their final year of primary school (11–12 years old) who completed a questionnaire during school hours. We examined the association between problematic social media use and psychosomatic complaints, as well as general life satisfaction and whether perceived social support and sex moderated these associations. Problematic social media use was associated with lower general life satisfaction, as well as all psychosomatic complaints, with the strongest association for having a bad mood or feeling irritated (OR = 3.08, 99% CI = 2.05–4.63). Most associations were not moderated by perceived social support or sex. Our findings indicate that the well-being of early adolescents may be affected by problematic social media use already in primary school. The association persisted regardless of the amount of perceived social support, and without strong gender differences. This suggests that the potential for limiting the potential negative consequences of problematic social media use through increasing social support is limited.

## 1. Introduction

Worldwide, social media use has rapidly increased over the past decade [1]. On average, people worldwide spend approximately 2.5 h per day on social media and use over seven different social media platforms each month. In the Netherlands, this trend is also visible, where social media are particularly popular among young people [2]. A clear generational divide in media use is evident, with traditional media such as printed newspapers and television seeing a decline [3], while high-visual social media platforms like Snapchat and Instagram are seeing increased usage among young people [4].

The increase in social media use is also reflected in the rise of problematic social media use in the Netherlands, especially among primary-school girls, which saw a more than twofold increase from 2017 to 2021 [5]. Problematic social media use characterizes individuals who experience addiction-like symptoms as a result of their social-media use [6]. It is different from excessive use of social media, typically defined solely on the basis of the hours of use [7]. Examples of detrimental effects that can occur as a result of preoccupation with and compulsion toward excessively engaging in social media platforms are feeling restless, stressed, or irritated when (not) having access to social media, or neglecting other tasks to spend more time on social media. In primary schools, 18.1% of eighth-grade students reported being in contact through social media throughout the day, and about 30% reported that they frequently could not think about anything other than their next social media session. Additionally, over 40% admitted to using social media often as a means of avoiding unpleasant thoughts, while 12.5% reported regularly having arguments with others about their social media usage [5].

Reasons for early adolescents to use social media are, for example, to connect with friends and family, fit in, feel happy, or reduce stress [8,9,10]. Although there are some positive associations with using social media, numerous studies also report on the downsides of social media use, since problematic social media use has been associated with worse mental health and well-being [11,12]. Possible explanations for this relationship include the risk of being cyberbullied; social comparison; observing triggering posts, and the prevalence of filtered images on social media platforms, which can be harmful for body image and self-esteem [13,14,15].

Despite extensive research on the association between social media use and mental health, several knowledge gaps remain. For example, the relationship with physical health complaints, such as having a headache or backache, has not been thoroughly explored [16,17]. For instance, poor posture while using social media on electronic devices, along with exposure to (blue) light, may lead to physical health issues. Additionally, if social media use negatively affects mental health, it may also have consequences for physical health [18]. Furthermore, a decline in mental and physical health could lead to reduced life satisfaction.

Given the frequent use of social media, gaining insights into these associations is crucial, especially since reported psychosomatic complaints among primary-school students have increased over the past years [5]. This raises the question whether the increase in social media use is a possible explanation for the increase in psychosomatic complaints. It is especially important to explore this relation in primary-school students, since habits are already formed at a young age [19], and if problematic social media use is related to adverse health consequences, this might also impact their health later in life [20]. Moreover, both mental and physical health are important for educational performance [21,22,23]. An unfavorable association with problematic social media use would not only have implications for early adolescents’ health, but likely also for their academic career.

Additionally, although the relationship between social media use and mental health has been examined and observed in many studies, there is a significant knowledge gap regarding factors that might influence the impact of social media use [24]. It is possible that the impact of social media use varies depending on individual characteristics, e.g., sex, as these might moderate its influence, resulting in varying effects for different people. Another moderating factor might be perceived social support, since evidence shows a negative relation between perceived social support and psychosomatic complaints [25,26]. For somatic complaints, the negative association with perceived social support is mainly explained by the mediating role of depressive symptoms and emotional self-efficacy [27]. The positive association between perceived social support and psychosocial health can be partly explained by better self-esteem [28]. Attachment theory research emphasizes the importance of emotional bonds between caregivers and their children, traditionally focusing on parent–child relationships. Teachers often serve as secondary attachment figures, providing emotional security and support when parents are absent. These attachment-like bonds with teachers can significantly contribute to children and young adolescents’ emotional and developmental outcomes, complementing the primary attachments formed with parents [29]. This connection between social support and both mental and physical health prompts the question whether social support could moderate the potential negative effects of social media use. Perceived high social support may serve as a buffer against the potential negative effects of social media use, so that early adolescents with strong social support may experience less psychosomatic complaints compared to those with low perceived social support.

A deeper understanding of these links is needed to equip schools, policymakers, and other stakeholders with relevant information, and to support them in decision-making regarding social media. Therefore, to contribute to the existing literature on the impact of social media use, we aimed to answer two research questions: (1) To what extent is problematic social media use related to well-being, operationalized as having psychosomatic complaints and general life satisfaction, in primary-school students in the Netherlands? (2) To what extent are these relationships moderated by perceived support from family, friends, teachers, and classmates, and sex at birth? We hypothesize that problematic social media use is negatively associated with well-being, but that the associations are weaker in students who perceive high social support from family, friends, classmates, or teachers. For sex at birth, we hypothesize that associations are stronger for girls.

## 2. Materials and Methods

This study took place as part of the Early Predictors of School Success (EPoSS) project. The study was approved by the Ethical Review Committee Inner City faculties of Maastricht University (ERCIC, ERCIC_503_16_11_2023).

### 2.1. Study Design, Study Population, Recruitment, and Data Collection

We conducted a cross-sectional study. Schools were recruited across the Netherlands from November 2023 to January 2024, with emails sent to all school boards nationwide. In the rare case where email addresses were unavailable, we sent a letter. School boards can oversee multiple schools, and the number of schools invited within each school board varied. Additionally, special-education schools, i.e., those that provide education to students with various disabilities, were excluded from participation. After a few weeks, a reminder email was sent. To enhance recruitment, we promoted the study on the National Educational Research Organisation (NRO)’s social media pages and created a website and flyer to inform school staff, parents, and students. 

Data collection took place between January and April 2024. A few weeks prior to data collection, parents and caregivers were notified by the school about the study and provided with an informed consent form, typically via email or a communication portal. Completed consent forms were sent directly to Maastricht University, which then notified the school of participating students. Within the participating schools, all students in their final year of primary education, i.e., eighth grade (around 11 or 12 years old), were able to participate if one parent or caregiver gave consent through digital IC forms. No reimbursements were offered to students, parents, or school staff for their participation. 

**Figure 1:**
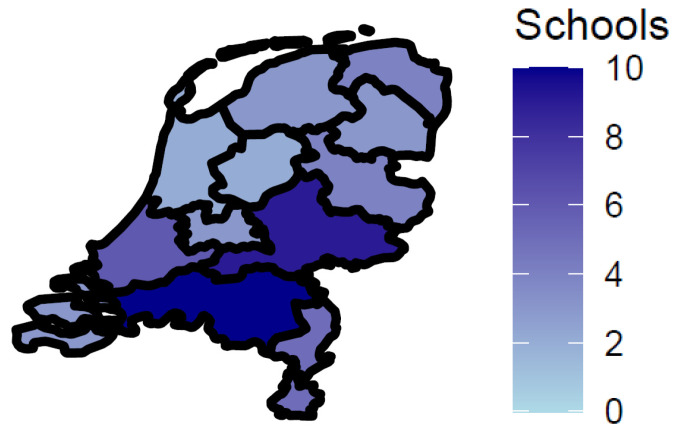
Number of participating schools per region in the Netherlands.

The students completed a digital questionnaire about their lifestyle and health during school hours. Researchers were not present. The median time students took to fill out the questionnaire was about 31 min. In total, we received 647 questionnaires. In case students filled out the questionnaire twice, we supplemented the data from the first questionnaire with information from the second questionnaire when applicable (N = 7). Additionally, we excluded students who did not give consent (N = 13), students without any data (N = 13), students who reported being in a different grade (N = 1), questionnaires filled out by (individuals assumed to be) teachers (N = 5), and students who could not be linked to the IC forms (N = 23). Finally, data from 585 primary-school students in their final year of primary education of 54 primary schools were included in our analysis. This was around 37% of the total invited population across all participating schools. As presented in Figure 1, we had a broad geographical coverage of the Netherlands and included schools in all twelve regions.

After data collection, encrypted data were securely stored at Statistics Netherlands (CBS). This allowed for individual-level linkage with data from the Netherlands Cohort Study on Education (NCO), using students’ sex, date of birth, and address. The NCO dataset provided information about students’ background characteristics (highest educational attainment of the parents, household income, and having a migration background) [30].

### 2.2. Measurements

#### 2.2.1. Problematic Social Media Use

A shortened version of the Compulsive Internet Use Scale (CIUS) was used to measure problematic social media use, where internet use was replaced with social media use [31]. Some questions were slightly adapted to make them more suitable for primary-school students. This scale included questions about having difficulties in reducing social media usage; external suggestions to spend less time on social media; preferring social media over in-person interactions; feeling restless, stressed, or irritated when not using social media or their phone; neglecting other tasks, like homework, in favor of social media; and using social media when feeling bad. For this study, we added two additional questions: one about using social media shortly before sleeping; and another about experiencing stress, irritation, or annoyance when using social media. Responses were rated on a 5-point scale, from never (0) to very often (4). The average score across all questions was calculated, with scores ranging from 0 to 4. Higher scores indicated higher problematic social media use. The internal consistency of the scale was tested for this study using Cronbach’s alpha, which was 0.83, indicating good consistency among the items.

#### 2.2.2. Outcomes

We examined the frequency of various psychosomatic complaints experienced by students in the six months preceding filling out the questionnaire. Psychosomatic complaints involve both psychological (i.e., mental health) and somatic (i.e., physical) complaints without a known cause or any assumptions about their origin. The term ‘psychosomatic’ underscores the interplay between the mind (psyche) and the body (soma) [32,33]. These complaints included having a headache, stomachache, backache, feelings of unhappiness, having bad moods or feeling irritated, feeling nervous, feeling dizzy, feeling nauseous, having concentration difficulties, having trouble falling asleep, sleeping bad, and experiencing daytime fatigue. For each complaint, we used a 5-point Likert scale with the following response options: ‘almost every day’, ‘more than once a week’, ‘almost every week’, ‘almost every month’, and ‘almost never or never’. We categorized these variables as binary outcomes (0/1), where a value of 1 indicated that the student experienced the psychosomatic complaint multiple times a week. The used question on psychosomatic complaints was created for the Health Behaviour in School-aged Children (HBSC) study, with some additional complaints [34,35]. The applied binary classification is also utilized by the HBSC study, but it is applied to all psychosomatic complaints collectively [5]. Since our goal was to explore the association between problematic social media use and individual psychosomatic complaints, we applied the classification to each complaint separately. Additionally, we assessed students’ general life satisfaction by asking them to rate their life on a scale from 0 to 10, where 0 represented the worst life imaginable, and 10 represented the best. This rating was included as a continuous outcome. General life satisfaction is a valuable variable for primary-school students since it captures well-being across a broad spectrum, from one extreme to the other [36].

#### 2.2.3. Potential Moderators

We examined perceived family support and support from friends as potential moderators in our study. Both variables were assessed through four statements, as used in the HBSC study as well, with slight adaptations [5]. Responses were rated on a scale from 1 (strongly disagree) to 7 (strongly agree). For family support, the statements evaluated whether family members really tried to help the student, whether the student received the necessary support and help at home, whether the student was able to talk about problems at home, and whether family members supported them in decision-making. If a student lived in multiple households, the questions were directed at the household where the student spent the majority of their time. Regarding friend support, the statements focused on whether friends genuinely tried to help the students, whether the students could rely on friends when things went wrong, whether they had friends with whom they could share everything, and whether they could talk with friends about their problems. The scores for each set of statements were averaged, and those above 5.5 were classified as high perceived social support from family and friends, consistent with the cut-off points used in the HBSC study [5].

We also included the relationship with teachers, as well as the dynamics among classmates. For both variables, three statements were provided using a 5-point Likert scale to measure responses, ranging from completely agree to completely disagree. Completely disagree was scored as 1, and completely agree as 5. The (slightly adapted) statements were also from the HBSC study [5]. The teacher-related questions evaluated whether students felt accepted by their teachers, whether they perceived their teachers cared about them, and whether they trusted their teachers. The questions regarding classmates assessed whether classmates enjoyed being together, whether students found their classmates to be friendly and helpful, and whether they felt accepted by their classmates. For each set of questions, total scores were calculated and divided by three to obtain an average score. Scores above 3.5 were classified as having a good relationship with teachers and experiencing good dynamics among classmates [5]. We will refer to these variables as support from teachers and support from classmates. Lastly, we also examined whether associations differed based on sex at birth, i.e., boys and girls.

#### 2.2.4. Covariates

The following covariates were included in our study: sex at birth (boy/girl, except when included as a moderator), having a migration background (yes, i.e., having at least one parent who was born outside the Netherlands/no), household income (low/medium or high), and highest educational attainment of the parents (low/medium or high). Information regarding the household income and parental education was obtained from the preliminary NCO dataset from school year 2021–2022, as this dataset offered the most recent available data. In case parents had different household incomes (e.g., because they are separated), we included the higher income for the analysis. Household incomes below the 75th percentile were categorized as low/medium, and household incomes at or above the 75th percentile were classified as high. The highest educational attainment of the parents was classified according to the Standard Education Classification (in Dutch, Standaardonderwijs Indeling) from Statistics Netherlands [37]. Information about migration background was also retrieved from the NCO dataset. We included all covariates dichotomously in our analysis model to prevent insufficient group sizes.

### 2.3. Statistical Analysis

RStudio version 4.4.0 was used to analyze the data [38]. Missing values (presented in Table A1 in Appendix A) were addressed through multiple imputations, with 10 imputations and 30 iterations. The mice package [39] was used to impute missing values using logistic regression and predictive mean matching. Following imputation, we conducted linear and logistic regression analyses to examine the relationship between problematic social media use and psychosomatic complaints, as well as general life satisfaction. To explore whether this relationship varied based on the level of perceived support from family, friends, teachers, and classmates, we included interaction terms between problematic social media use and perceived support variables in separate models. For significant interactions (*p* < 0.1), stratified analyses were conducted. All analyses were adjusted for covariates, as detailed in the covariates section. For logistic regression analyses, we calculated Odds Ratios (ORs). We reported 99% confidence intervals and whether *p*-values were below 0.1, 0.05, and 0.01.

## 3. Results

### 3.1. Descriptive Statistics

**Figure 2:**
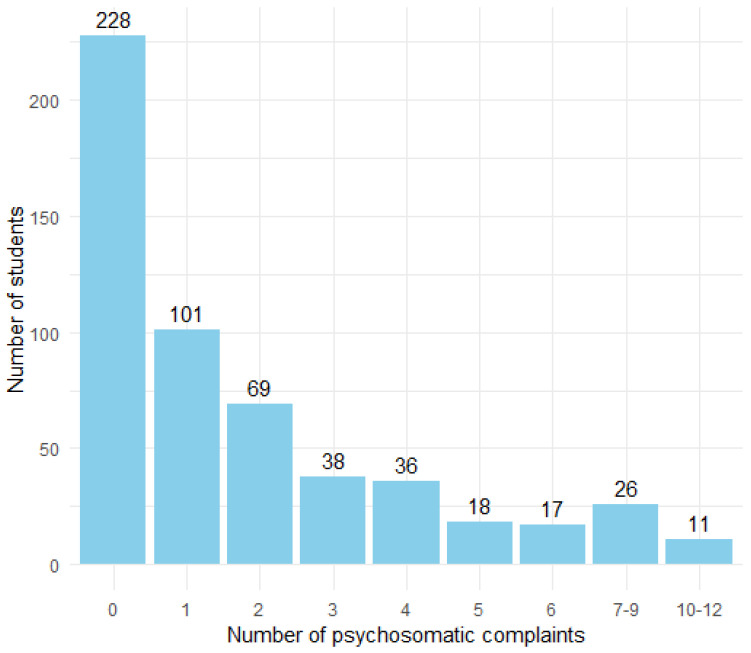
The number of students by complaint count. Note: A student was considered to have a complaint if they reported experiencing it multiple times a week. We included data of 544 students; 41 students had one or more missing values for the psychosomatic complaints.

For the current study, data of 585 eighth-grade students were analyzed. Within our study sample of 585 students, 49.3% of them were born a boy, 13.7% had a migration background, 63.0% had a high household income, and 70.0% had at least one parent with high educational attainment. Students reported on average a score of 1.34 for problematic social media use on a scale ranging from 0 to 4. For the psychosomatic complaints, the percentage of students reporting symptoms multiple times a week varies by complaint, ranging from 7.6% to 30.5%. The psychosomatic complaints that were reported most often to occur multiple times a week were having trouble falling asleep (30.5%), having concentration difficulties (24.6%), and experiencing daytime fatigue (23.6%). Psychosomatic complaints that were reported least often were feeling nauseous (7.6%), having a backache (8.1%), and feeling dizzy (8.8%). In Figure 2, we present an overview of the number of students by complaint count. In total, 41.9% reported having 0 complaints multiple times a week, 38.2% reported having 1–3 complaints multiple times a week, and 19.9% reported having more than 3 complaints multiple times a week. Additionally, for support, 89.7% of students perceived support from their family to be high, while for friends, this was 66.2%. Support from teachers was perceived as high by 86.3% of students, and 80.8% experienced high support from classmates.

Background characteristics did not differ between boys and girls (Table 1). However, girls scored higher on problematic social media use (1.44 vs. 1.24, *p* < 0.01) and graded their general life satisfaction lower (7.71 vs. 8.19, *p* < 0.01). For several psychosomatic complaints, girls more often reported experiencing them multiple times a week, i.e., having a headache (16.2% vs. 8.7%, *p* = 0.02), having a stomachache (14.9% vs. 6.1%, *p* ≤ 0.01), feeling unhappy (12.9% vs. 7.6%, *p* = 0.05), having a bad mood or feeling irritated (25.3% vs. 15.6%, *p* < 0.01), feeling nervous (18.3% vs. 9.7%, *p* < 0.01), feeling dizzy (12.5% vs. 5.1%, *p* < 0.01), feeling nauseous (10.5% vs. 4.7%, *p* = 0.02), and having trouble falling asleep (33.6% vs. 26.3%, *p* = 0.07). Additionally, girls perceived support from friends more often as high compared to boys (72.2% vs. 59.9%, *p* < 0.01). An overview of the missing values is presented in Table A1 in Appendix A.

### 3.2. Association Between Problematic Social Media Use and Well-Being

**Figure 3:**
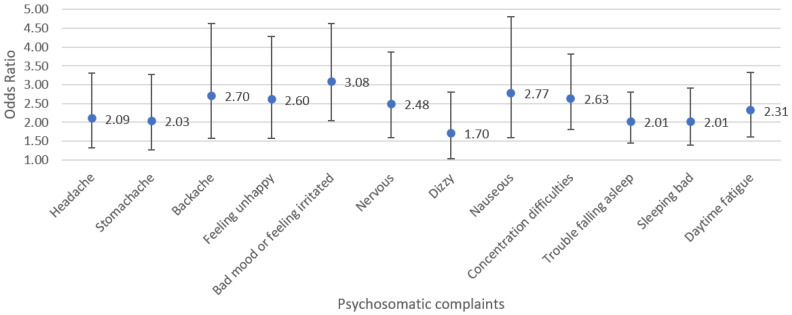
The association between problematic social media use and psychosomatic complaints (N = 585). Note: Problematic social media use is included as a continuous variable, ranging from 0 to 4. Odds ratios are presented for having the psychosomatic complaint multiple times a week, including the 99% confidence interval (CI). All analyses are adjusted for covariates, i.e., sex, household income, highest educational attainment of the parents, and having a migration background. The parameters for these covariates are not presented. *p*-values were below 0.01 for all outcomes.

We examined the association between problematic social media use and having psychosomatic complaints multiple times a week, for every complaint separately. Results are presented in Figure 3. ORs ranged from 1.70 to 3.08, indicating that students who score on average one point higher on the social media scale are approximately two to three times as likely to experience the psychosomatic complaint. The strongest associations were identified for having a bad mood or feeling irritated (OR = 3.08, 99% CI = 2.05–4.63), feeling nauseous (OR = 2.77, 99% CI = 1.60–4.80), having a backache (OR = 2.70, 99% CI = 1.57–4.63), and having concentration difficulties (OR = 2.63, 99% CI = 1.81–3.82). For general life satisfaction, we observed a significant negative association (B = −0.65, 99% CI = −0.85; −0.44, *p* < 0.01) with problematic social media use.

### 3.3. Moderation by Perceived Social Support and Sex

In the next step, we examined whether the observed associations between problematic social media use and psychosomatic complaints, as well as general life satisfaction, differed for students who perceived social support as high compared to low. An overview of all results is presented in Table A2, Table A3, Table A4, Table A5, Table A6 and Table A7 in Appendix A. For significant interaction effects, stratified analyses are presented in Table 2 and Table 3. Perceived social support did not moderate the association for most outcomes, except for a few: Perceiving support from family as low weakened the association between problematic social media use and having a headache (high family support: OR = 2.35 (1.40–3.94); low family support: OR = 1.11 (0.43–2.88)), having a bad mood or feeling irritated (high family support: OR = 3.61 (2.24–5.84); low family support: OR = 1.51 (0.57–4.00)), and experiencing daytime fatigue (high family support: OR = 2.49 (1.63–3.80); low family support: OR = 1.22 (0.54–2.77)). Sex moderated the associations between problematic social media use and having trouble falling asleep. We observed a stronger association for girls compared to boys (boys: OR = 1.48; girls: OR = 2.65).

## 4. Discussion

The aim of the current study was to examine to what extent problematic social media use is related to psychosomatic complaints and general life satisfaction among primary-school students in the Netherlands, and whether the association differs depending on perceived social support. We found that problematic social media use was associated with all included outcomes, i.e., having a headache, stomachache, backache, feeling unhappy, having a bad mood or feeling irritated, feeling nervous, feeling dizzy, feeling nauseous, having concentration difficulties, having trouble falling asleep, sleeping bad, daytime fatigue, and general life satisfaction. 

The ORs ranged from 1.70 for feeling dizzy to 3.08 for having a bad mood or feeling irritated, indicating that there were small-to-medium associations [40]. This indicates that students who score one point higher on the scale for problematic social media use are more than three times as likely to report having a bad mood or feeling irritated multiple times a week. Overall, it appears that students who score higher on problematic social media use have both lower mental and physical health. These results are particularly concerning when considering the young age of our study population. Childhood is an important period for habit formation and learning [19], and health conditions experienced during this stage can track into adulthood [20]. Moreover, it was noteworthy that for the complaints that were least frequently reported, i.e., feeling nauseous and having a backache, we identified the strongest association with problematic social media use, besides having a bad mood or feeling irritated. While causal mechanisms cannot be implied from our analyses, it is worth exploring in future studies whether problematic social media use might indeed contribute to the occurrence of rare psychosomatic complaints in this age group. 

Our findings are consistent with the existing literature. Previous studies also identified significant negative associations between social media use and multiple outcomes, such as mental health, sleep quality, and headaches [17,41]. While the study of Alonzo et al. [41] focused on youth aged 16 years and older, our study confirms that social media use is also associated with psychosomatic complaints in younger age groups, and this finding has implications for prevention policies. The review by Bozzola et al. [18] included mostly studies that focused on mental health, while only three out of the sixty-eight included studies included headache as an outcome. One of the explanations for having more trouble falling asleep and sleeping bad is that using electronic devices before bedtime can negatively influence the production of melatonin [42]. Sleep disturbance can in turn cause daytime fatigue, but is also related to multiple mental health disorders, such as depression [43]. Additionally, many social media platforms are designed to promote addictive behavior [44], therefore probably causing students to spend more time on social media than intended. This extended time spent on social media, combined with improper posture during the use of devices to use social media might cause backaches and headaches [45]. A qualitative study among adolescents with depression highlighted the potential risks of social media for worsening well-being. Factors that were discussed were observing positive posts of others, e.g., seeing others hanging out or having fun together; social comparison; triggering posts; or the risk of oversharing, which may lead to being cyberbullied or feeling embarrassed [14].

It is noticeable that unfavorable associations were observed between problematic social media use and all included psychosomatic complaints, as well as general life satisfaction. It is possible that these psychosomatic complaints are interrelated and may causally influence each other. For example, early adolescents who more often experience stomachaches or headaches might more often have difficulties falling asleep because of the pain, causing them to feel more tired during the day and have more concentration difficulties. In addition, sleep is also important for the regulation of emotions, as a deficit can cause more negative emotions [46]. It is possible that the lower observed general life satisfaction is a consequence of the more frequent occurrence of physical and mental health complaints, but it is also possible that problematic social media use has a direct influence on life satisfaction, for example, due to social comparison. Future research should examine whether the association between problematic social media use and general life satisfaction is mediated by decreased health.

Besides the observed mental and physical health concerns of problematic social media use, these psychosomatic complaints might also have a negative influence on students’ academic careers. Students who experience more physical health problems and have lower well-being, as well as students who have more issues with concentration and have worse sleep quality, perform generally worse in school [47,48,49,50]. For example, a recent study from the Programme of International Student Assessment (PISA) shows that educational performance of Dutch secondary school students has decreased over the past decade, especially for reading skills [51]. During this period, (problematic) social media use has strongly increased [2].

Another aim of the current study was to examine whether this association can differ depending on the level of perceived social support. Although a few associations were moderated by social support, most outcomes showed no significant differences in associations between students who perceive social support as high or low. This shows how concerning the influence of social media use may be, since associations appear to be present for eighth-grade students, regardless of their social support. Nevertheless, a few associations were moderated by perceived social support from family, which appeared more important for influencing the associations than support from teachers, friends, and classmates. The association between problematic social media use and having a headache, having a bad mood or feeling irritated, and experiencing daytime fatigue weakened for students who perceived social support from family to be low. This was not in line with our hypothesis. Our results showed that students with low family support experience psychosomatic complaints more often in general. It is possible that early adolescents who have less supportive home environments already face concerns that may affect their well-being, while early adolescents with high family support have more room to be negatively affected by their problematic social media use. It is also possible that social media provides early adolescents with low family support a means to escape from their home environment and seek online support from peers and friends. Additionally, our findings showed that girls reported lower general life satisfaction and more often had psychosomatic complaints compared to boys. This was in line with the existing literature [5]. However, associations between problematic social media use and these psychosomatic complaints were not stronger for girls, except for having trouble falling asleep.

The findings of our study indicate how concerning the popularity of social media among primary-school students is. Although more research is needed to examine the directionality, increased awareness among students and their parents is needed about the potential risks of problematic social media use for these students. Even though most popular social media platforms have age restrictions, i.e., prohibiting adolescents under 13 from creating accounts [9], a study by CBS from 2019 revealed that nearly the entire population in the Netherlands aged 12 to 45 years old uses social media [3]. Despite the existence of age restrictions, it is very easy to create an account if you are too young according to user guidelines. Because of the concerning associations with many physical and mental health complaints, supervision on the apps should be strengthened. Additionally, awareness of the potential risks of social media use needs to be raised among parents. Early adolescents should also be educated about the potential dangers of social media use, potentially through schools. For instance, the Dutch Healthy School program, which is widely adopted across schools in the Netherlands, also provides support to schools in stimulating responsible media use among primary-school students [52]. Additionally, governments can play a crucial role by implementing laws aimed at reducing addictive features and mechanisms built into social media platforms, further protecting young users from harm. For example, since the school year 2024–2025, phones have been prohibited during classes in primary schools in the Netherlands. This might reduce (problematic) the social media use of students.

### Limitations and Directions for Future Research

The current study contributed to the existing literature on the association between problematic social media use and health indicators, while also examining the potential influence of perceived social support and sex differences. Moreover, our study had good geographical coverage of schools in the Netherlands. A notable strength of the current study was the use of recent data from eighth-grade students, while including a broad range of outcomes. In particular, the association with physical health complaints has not been studied to a great extent to this date, as well as the moderation by individual and contextual factors. Therefore, this study provides timely insights into a rapidly evolving topic. 

However, the current study also had several limitations that should be taken into account. One limitation was its cross-sectional design, which restricts the ability to draw conclusions about causality and the direction of relationships between variables. For example, it is possible that early adolescents who have more difficulties falling asleep have a higher problematic social media use, instead of the other way around. Future studies should explore directionality. Moreover, we had to obtain active, informed consent from parents or caregivers. Therefore, we only included data of around 37% of invited students, and doing so potentially resulted in selection bias. Our study sample had relatively fewer students with a migration background and parents with a low/medium educational attainment compared to the total study population within the participating schools, as well as all eighth-grade students in the Netherlands. For this comparison, we used preliminary data of eighth-grade students in 2021–2022, since these were the most recent available data. The difference between our study sample and the total study population might be a consequence of using solely Dutch recruitment materials and only providing a Dutch questionnaire, as well as an extensive IC-form. Therefore, we assume the generalizability to early adolescents from lower-SES families and early adolescents with a migration background to be limited. To increase the number of participants, future studies could use recruitment materials or questionnaires in different languages, or use different recruitment strategies, such as verbal, instead of written, recruitment. Additionally, students participating in our study also completed an end-of-primary-school test, with the data expected to be available in 2025. This presents an immediate opportunity to examine whether problematic social media use is indeed associated with lower educational performance. Based on the current findings, we hypothesize that problematic social media use is related to both lower well-being and worse educational performance.

## 5. Conclusions

Our findings indicate that among early adolescents in the final year of primary school, problematic social media use is unfavorably associated with a variety of psychosomatic complaints, as well as general life satisfaction, indicating that well-being may be affected by social media use. These results are particularly worrying given the young age of our study population. The association between problematic social media use and well-being persisted regardless of the amount of perceived social support, and without consistent differences between boys and girls. Given the well-established association between well-being and educational performance, our findings indicate that problematic social media use might interfere with educational performance. Stakeholders, such as students, parents, schools, and policymakers, should be informed about the potential risks of social media use.

## Figures and Tables

**Table 1:** Descriptive statistics of a subsample of Dutch primary-school students in the eighth grade (N = 585).

Variable	Total (N = 585 ^1^)	Boys (N = 286)	Girls (N = 294)
Age in years (mean (SD))	11.4 (0.51)	11.4 (0.51)	11.3 (0.52)
Having a migration background (%)	13.7	14.8	12.8
Household income (% high)	63.0	59.3	66.3
Highest parental educational attainment (% high)	68.3	68.5	68.0
Problematic social media use (mean (SD); range 0–4)	1.34 (0.77)	1.24 (0.75)	1.44 (0.78) ***
Psychosomatic complaints (% more than weekly)			
Headache	12.4	8.7	16.2 ***
Stomachache	10.7	6.1	14.9 ***
Backache	8.1	6.5	9.4
Feeling unhappy	10.3	7.6	12.9 *
Having a bad mood or feeling irritated	20.6	15.6	25.3 ***
Feeling nervous	14.1	9.7	18.3 ***
Feeling dizzy	8.8	5.1	12.5 ***
Feeling nauseous	7.6	4.7	10.5 **
Having concentration difficulties	24.6	21.7	26.4
Having trouble falling asleep	30.5	26.3	33.6 *
Sleeping bad	20.0	18.4	20.8
Experiencing daytime fatigue	23.6	21.4	24.7
General life satisfaction (mean (SD); range 1–10)	7.94 (1.5)	8.19 (1.37)	7.71 (1.51) ***
Family support (% high)	89.6	89.3	90.2
Support from friends (% high)	66.2	59.9	72.2 ***
Teacher support (% high)	86.3	84.6	88.2
Support from classmates (% high)	80.8	83.6	78.3

^1^ There were five missing values for sex at birth. Comparison between boys and girls: * *p* < 0.1, ** *p* < 0.05, and *** *p* < 0.01.

**Table 2:** Stratified analysis for the role of social support in moderating the association between problematic social media use and psychosomatic complaints (N = 585).

		Headache	Having a Bad Mood or Feeling Irritated	Experiencing Daytime Fatigue
		OR (99% CI)		
Family support (high)	Intercept	0.02 (0.01–0.07) ***	0.03 (0.01–0.08) ***	0.07 (0.03–0.15) ***
	Problematic social media use	2.35 (1.40–3.94) ***	3.61 (2.24–5.84) ***	2.49 (1.63–3.80) ***
Family support (low)	Intercept	0.15 (0.01–1.58) **	0.08 (0.01–1.16)	0.56 (0.08–3.72)
	Problematic social media use	1.11 (0.43–2.88)	1.51 (0.57–4.00)	1.22 (0.54–2.77)

Note: Problematic social media use is included as a continuous variable, ranging from 0 to 4. The outcomes are included as binary variables, where a value of 1 indicates that the student experienced the psychosomatic complaint multiple times a week. All analyses are adjusted for covariates, i.e., sex, household income, highest educational attainment of the parents, and having a migration background. The parameters for these covariates are not presented. ** *p*-value < 0.05, and *** *p*-value < 0.01. Results are presented as Odds Ratios (ORs), including the 99% confidence interval (CI).

**Table 3:** Stratified analyses for the role of sex in moderating the association between problematic social media use and psychosomatic complaints (N = 585).

		Having Trouble Falling Asleep
		B (99% CI)
Boys	Intercept	0.24 (0.11–0.55) ***
	Problematic social media use	1.48 (0.90–2.43) **
Girls	Intercept	0.11 (0.04–0.26) ***
	Problematic social media use	2.65 (1.63–4.31) ***

Note: Problematic social media use is included as a continuous variable, ranging from 0 to 4. The outcome is included as a binary variable, where a value of 1 indicates that the student experienced the psychosomatic complaint multiple times a week. The analysis is adjusted for covariates, i.e., household income, highest educational attainment of the parents, and having a migration background. The parameters for these covariates are not presented. ** *p*-value < 0.05, and *** *p*-value < 0.01. Results are presented as Odds Ratios (ORs), including the 99% confidence interval (CI).

## Data Availability

Restrictions apply to the availability of these data. The data supporting the conclusions of this article can be made available by the authors upon request, under strict conditions. Data from the Netherlands Cohort Study on Education (NCO) regarding socioeconomic status and migration background were obtained from the National Educational Research Organisation’s (NRO), available at Statistics Netherlands (CBS), also under strict conditions.

## Appendix A

**Table A1:** An overview of the missing values ^1^.

Variable	Total (N = 585) ^2^	Boys (N = 286)	Girls (N = 294)
Having a migration background	88 (15.0%)	43 (15.0%)	44 (15%)
Household income	91 (15.6%)	45 (15.7%)	45 (15.3%)
Highest parental educational attainment	137 (23.4%)	67 (23.4%)	69 (23.5%)
Problematic social media use	37 (6.3%)	18 (6.3%)	19 (6.5%)
Psychosomatic complaints			
Headache	13 (2.2%)	9 (3.1%)	4 (1.4%)
Stomachache	13 (2.2%)	8 (2.8%)	5 (1.7%)
Backache	17 (2.9%)	9 (3.1%)	8 (2.7%)
Feeling unhappy	24 (4.1%)	9 (3.1%)	15 (5.1%)
Having a bad mood or feeling irritated	16 (2.7%)	10 (3.5%)	6 (2.0%)
Feeling nervous	19	9 (3.1%)	10 (3.4%)
Feeling dizzy	16 (2.7%)	9 (3.1%)	7 (2.4%)
Feeling nauseous	16 (2.7%)	9 (3.1%)	7 (2.4%)
Having concentration difficulties	16 (2.7%)	10 (3.5%)	6 (2.0%)
Having trouble falling asleep	17 (2.9%)	8 (2.8%)	8 (2.7%)
Sleeping bad	20 (3.4%)	9 (3.1%)	11 (3.7%)
Experiencing daytime fatigue	17 (2.9%)	10 (3.5%)	7 (2.4%)
General life satisfaction	23 (3.9%)	10 (3.5%)	12 (4.1%)
Family support	15 (2.6%)	6 (2.1%)	9 (3.1%)
Support from friends	17 (2.9%)	7 (2.4%)	9 (3.1%)
Teacher support	37 (6.3%)	20 (7.0%)	15 (5.1%)
Support from classmates	43 (7.4%)	24 (8.4%)	18 (6.1%)

^1^ Missing values also include the answer category ‘I’d rather not say’. ^2^ There were five missing values for sex at birth.

**Table A2:** The role of family support in moderating the association between problematic social media use and psychosomatic complaints (N = 585).

	Headache		Stomachache		Backache		Feeling Unhappy	
	OR	99% CI	OR	99% CI	OR	99% CI	OR	99% CI
Intercept	0.02	(0.01–0.07) ***	0.03	(0.01–0.08) ***	0.01	(0.00–0.04) ***	0.02	(0.00–0.06) ***
Problematic social media use	2.32	1.39–3.88) ***	2.09	(1.23–3.56) ***	3.10	(1.73–5.58) ***	2.55	(1.43–4.55) ***
Family support (low)	9.28	(1.31–65.76) ***	2.15	(0.15–30.19)	3.19	(0.15–69.39)	6.88	(0.63–75.13) **
Social media use × family support (low)	0.47	(0.17–1.29) *	0.75	(0.22–2.59)	0.49	(0.11–2.10)	0.71	(0.22–2.33)
	Having a bad mood or feeling irritated		Feeling nervous		Feeling dizzy		Feeling nauseous	
	OR	99% CI	OR	99% CI	OR	99% CI	OR	99% CI
Intercept	0.03	(0.01–0.07) ***	0.03	(0.01–0.07) ***	0.02	(0.01–0.07) ***	0.01	(0.00–0.04) ***
Problematic social media use	3.58	(2.23–5.75) ***	2.51	(1.55–4.08) ***	1.69	(0.97–2.96) **	2.67	(1.44–4.93) ***
Family support (low)	5.72	(0.75–43.79) **	2.76	(0.30–25.70)	0.92	(0.05–18.64)	1.40	(0.06–33.36)
Social media use × family support (low)	0.42	(0.15–1.20) **	0.76	(0.26–2.21)	1.04	(0.26–4.13)	1.02	(0.26–4.07)
	Having concentration difficulties		Having trouble falling asleep		Sleeping bad		Experiencing daytime fatigue	
	OR	99% CI	OR	99% CI	OR	99% CI	OR	99% CI
Intercept	0.09	(0.04–0.18) ***	0.14	(0.07–0.27) ***	0.08	(0.04–0.17) ***	0.07	(0.03–0.15) ***
Problematic social media use	2.49	(1.65–3.77) ***	1.95	(1.35–2.82) ***	2.09	(1.37–3.19) ***	2.48	(1.63–3.77) ***
Family support (low)	1.04	(0.10–10.64)	2.60	(0.45–15.04)	9.06	(1.52–53.96) ***	7.08	(1.29–38.83) ***
Social media use × family support (low)	1.19	(0.38–3.74)	0.88	(0.34–2.29)	0.56	(0.22–1.39)	0.51	(0.21–1.25) *

Note: Problematic social media use is included as a continuous variable, ranging from 0 to 4. The outcomes are included as binary variables, where a value of 1 indicates that the student experienced the psychosomatic complaint multiple times a week. All analyses are adjusted for covariates, i.e., sex, household income, highest educational attainment of the parents, and having a migration background. The parameters for these covariates are not presented. * *p*-value < 0.1, ** *p*-value < 0.05, and *** *p*-value < 0.01. Results are presented as Odds Ratios (ORs), including the 99% confidence interval (CI).

**Table A3:** The role of support from friends in moderating the association between problematic social media use and psychosomatic complaints (N = 585).

	Headache		Stomachache		Backache		Feeling Unhappy	
	OR	99% CI	OR	99% CI	OR	99% CI	OR	99% CI
Intercept	0.03	(0.01–0.10) ***	0.03	(0.01–0.09) ***	0.02	(0.00–0.07) ***	0.01	(0.00–0.06) ***
Problematic social media use	2.17	(1.23–3.81) ***	1.86	(1.01–3.43) ***	2.18	(1.10–4.33) ***	3.02	(1.51–6.03) ***
Support from friends (low)	1.06	(0.19–5.97)	1.08	(0.19–6.27)	0.55	(0.05–6.50)	3.68	(0.54–25.16) *
Social media use × support from friends (low)	0.92	(0.37–2.28)	1.17	(0.46–3.00)	1.65	(0.49–5.56)	0.67	(0.25–1.79)
	Having a bad mood or feeling irritated		Feeling nervous		Feeling dizzy		Feeling nauseous	
	OR	99% CI	OR	99% CI	OR	99% CI	OR	99% CI
Intercept	0.03	(0.01–0.08) ***	0.01	(0.00–0.05) ***	0.02	(0.00–0.06) ***	0.01	(0.00–0.04) ***
Problematic social media use	3.38	(1.97–5.80) ***	2.71	(1.42–5.15) ***	1.87	(0.99–3.51) **	3.17	(1.65–6.12) ***
Support from friends (low)	2.11	(0.41–10.80)	4.59	(0.90–23.50) **	1.97	(0.27–14.16)	1.66	(0.16–17.58)
Social media use × support from friends (low)	0.76	(0.31–1.85)	0.76	(0.31–1.83)	0.76	(0.25–2.27)	0.70	(0.21–2.28)
	Having concentration difficulties		Having trouble falling asleep		Sleeping bad		Experiencing daytime fatigue	
	OR	99% CI	OR	99% CI	OR	99% CI	OR	99% CI
Intercept	0.07	(0.03–0.16) ***	0.15	(0.07–0.31) ***	0.10	(0.04–0.23) ***	0.08	(0.03–0.18) ***
Problematic social media use	2.73	(1.68–4.43) ***	1.79	(1.18–2.71) ***	1.83	(1.15–2.92) ***	2.28	(1.41–3.69) ***
Support from friends (low)	1.74	(0.47–6.46)	0.82	(0.24–2.78)	0.97	(0.23–4.01)	1.31	(0.35–4.93)
Social media use × support from friends (low)	0.86	(0.41–1.83)	1.31	(0.64–2.68)	1.21	(0.55–2.68)	0.99	(0.46–2.11)

Note: Problematic social media use is included as a continuous variable, ranging from 0 to 4. The outcomes are included as binary variables, where a value of 1 indicates that the student experienced the psychosomatic complaint multiple times a week. All analyses are adjusted for covariates, i.e., sex, household income, highest educational attainment of the parents, and having a migration background. The parameters for these covariates are not presented. * *p*-value < 0.1, ** *p*-value < 0.05, and *** *p*-value < 0.01. Results are presented as Odds Ratios (ORs), including the 99% confidence interval (CI).

**Table A4:** The role of teacher support in moderating the association between problematic social media use and psychosomatic complaints (N = 585).

	Headache		Stomachache		Backache		Feeling Unhappy	
	OR	99% CI	OR	99% CI	OR	99% CI	OR	99% CI
Intercept	0.03	(0.01–0.09) ***	0.03	(0.01–0.08) ***	0.01	(0.00–0.04) ***	0.02	(0.01–0.06) ***
Problematic social media use	2.10	(1.25–3.53) ***	1.91	(1.12–3.28) ***	2.98	(1.60–5.56) ***	2.65	(1.54–4.58) ***
Teacher support (low)	1.67	(0.18–15.90)	1.49	(0.10–21.15)	4.16	(0.30–57.76)	1.46	(0.06–33.74)
Social media use × teacher support (low)	0.87	(0.29–2.63)	1.05	(0.27–4.07)	0.55	(0.15–2.02)	0.87	(0.19–3.87)
	Having a bad mood or feeling irritated		Feeling nervous		Feeling dizzy		Feeling nauseous	
	OR	99% CI	OR	99% CI	OR	99% CI	OR	99% CI
Intercept	0.03	(0.01–0.08) ***	0.04	(0.01–0.10) ***	0.02	(0.00–0.05) ***	0.01	(0.00–0.04) ***
Problematic social media use	2.99	(1.87–4.77) ***	2.24	(1.37–3.69) ***	1.90	(1.07–3.39) ***	3.08	(1.63–5.80) ***
Teacher support (low)	1.39	(0.16–11.84)	0.11	(0.00–11.19)	5.33	(0.56–50.47) *	2.92	(0.14–62.14)
Social media use × teacher support (low)	1.00	(0.34–2.96)	2.62	(0.36–18.97)	0.51	(0.15–1.67)	0.58	(0.13–2.46)
	Having concentration difficulties		Having trouble falling asleep		Sleeping bad		Experiencing daytime fatigue	
	OR	99% CI	OR	99% CI	OR	99% CI	OR	99% CI
Intercept	0.07	(0.03–0.15) ***	0.14	(0.07–0.27) ***	0.10	(0.05–0.21) ***	0.07	(0.03–0.16) ***
Problematic social media use	2.65	(1.73–4.06) ***	1.92	(1.32–2.79) ***	1.92	(1.25–2.93) ***	2.28	(1.52–3.44) ***
Teacher support (low)	3.40	(0.64–17.97) *	1.36	(0.25–7.48)	0.96	(0.11–8.71)	2.60	(0.49–13.82)
Social media use × teacher support (low)	0.74	(0.30–1.78)	1.06	(0.43–2.62)	1.16	(0.40–3.35)	0.82	(0.34–1.97)

Note: Problematic social media use is included as a continuous variable, ranging from 0 to 4. The outcomes are included as binary variables, where a value of 1 indicates that the student experienced the psychosomatic complaint multiple times a week. All analyses are adjusted for covariates, i.e., sex, household income, highest educational attainment of the parents, and having a migration background. The parameters for these covariates are not presented. * *p*-value < 0.1 and *** *p*-value < 0.01. Results are presented as Odds Ratios (ORs), including the 99% confidence interval (CI).

**Table A5:** The role of support from classmates in moderating the association between problematic social media use and psychosomatic complaints (N = 585).

	Headache		Stomachache		Backache		Feeling Unhappy	
	OR	99% CI	OR	99% CI	OR	99% CI	OR	99% CI
Intercept	0.03	(0.01–0.10) ***	0.04	(0.01–0.11) ***	0.01	(0.00–0.06) ***	0.01	(0.00–0.06) ***
Problematic social media use	1.96	(1.09–3.55) ***	1.58	(0.89–2.80) **	2.60	(1.29–5.23) ***	2.32	(1.15–4.70) ***
Support from classmates (low)	1.38	(0.22–8.73)	0.39	(0.04–3.88)	1.29	(0.11–15.20)	6.48	(0.97–43.32) **
Social media use × support from classmates (low)	1.05	(0.42–2.67)	1.95	(0.66–5.76)	1.01	(0.32–3.15)	0.93	(0.35–2.48)
	Having a bad mood or feeling irritated		Feeling nervous		Feeling dizzy		Feeling nauseous	
	OR	99% CI	OR	99% CI	OR	99% CI	OR	99% CI
Intercept	0.03	(0.01–0.09) ***	0.02	(0.01–0.07) ***	0.02	(0.01–0.07) ***	0.01	(0.00–0.04) ***
Problematic social media use	2.72	(1.65–4.47) ***	2.61	(1.47–4.62) ***	1.73	(0.93–3.20) **	3.00	(1.49–6.04) ***
Support from classmates (low)	2.35	(0.46–12.05)	3.45	(0.62–19.35) *	1.37	(0.16–11.49)	2.37	(0.20–27.61)
Social media use × support from classmates (low)	1.15	(0.47–2.82)	0.73	(0.30–1.79)	0.91	(0.30–2.75)	0.73	(0.24–2.30)
	Having concentration difficulties		Having trouble falling asleep		Sleeping bad		Experiencing daytime fatigue	
	OR	99% CI	OR	99% CI	OR	99% CI	OR	99% CI
Intercept	0.07	(0.03–0.16) ***	0.16	(0.08–0.30) ***	0.10	(0.04–0.22) ***	0.08	(0.03–0.17) ***
Problematic social media use	2.56	(1.63–4.03) ***	1.81	(1.22–2.69) ***	1.88	(1.19–2.98) ***	2.17	(1.39–3.38) ***
Support from classmates (low)	3.23	(0.79–13.21) **	0.90	(0.22–3.73)	1.27	(0.26–6.20)	2.30	(0.55–9.53)
Social media use × support from classmates (low)	0.88	(0.41–1.90)	1.31	(0.60–2.83)	1.09	(0.48–2.48)	0.99	(0.46–2.13)

Note: Problematic social media use is included as a continuous variable, ranging from 0 to 4. The outcomes are included as binary variables, where a value of 1 indicated that the student experienced the psychosomatic complaint multiple times a week. All analyses are adjusted for covariates, i.e., sex, household income, highest educational attainment of the parents, and having a migration background. The parameters for these covariates are not presented. * *p*-value < 0.1, ** *p*-value < 0.05, and *** *p*-value < 0.01. Results are presented as Odds Ratios (ORs), including the 99% confidence interval (CI).

**Table A6:** The role of sex in moderating the association between problematic social media use and psychosomatic complaints (N = 585).

	Headache		Stomachache		Backache		Feeling Unhappy	
	OR	99% CI	OR	99% CI	OR	99% CI	OR	99% CI
Intercept	0.03	(0.01–0.13) ***	0.03	(0.01–0.13) ***	0.01	(0.00–0.07) ***	0.03	(0.01–0.11) ***
Problematic social media use	2.03	(0.94–4.39) **	2.05	(0.89–4.71) **	2.72	(1.19–6.23) ***	2.34	(1.09–5.02) ***
Sex (girl)	1.59	(0.30–8.48)	2.35	(0.37–14.95)	1.28	(0.16–10.24)	1.08	(0.17–6.69)
Social media use × sex (girl)	1.05	(0.42–2.62)	0.99	(0.36–2.70)	0.98	(0.34–2.84)	1.20	(0.45–3.16)
	Having a bad mood or feeling irritated		Feeling nervous		Feeling dizzy		Feeling nauseous	
	OR	99% CI	OR	99% CI	OR	99% CI	OR	99% CI
Intercept	0.03	(0.01–0.12) ***	0.04	(0.01–0.13) ***	0.03	(0.01–0.15) ***	0.01	(0.00–0.08) ***
Problematic social media use	3.07	(1.63–5.79) ***	2.19	(1.12–4.28) ***	1.33	(0.52–3.37)	2.47	(0.98–6.24) **
Sex (girl)	1.53	(0.34–6.90)	1.20	(0.24–6.08)	1.38	(0.18–10.51)	1.36	(0.13–14.08)
Social media use × sex (girl)	1.01	(0.44–2.31)	1.23	(0.51–2.95)	1.43	(0.44–4.68)	1.20	(0.37–3.92)
	Having concentration difficulties		Having trouble falling asleep		Sleeping bad		Experiencing daytime fatigue	
	OR	99% CI	OR	99% CI	OR	99% CI	OR	99% CI
Intercept	0.07	(0.03–0.19) ***	0.22	(0.10–0.48) ***	0.14	(0.06–0.33) ***	0.08	(0.03–0.20) ***
Problematic social media use	2.89	(1.66–5.02) ***	1.46	(0.89–2.41) *	1.57	(0.91–2.72) **	2.44	(1.42–4.20) ***
Sex (girl)	1.39	(0.38–5.09)	0.51	(0.16–1.62)	0.47	(0.13–1.73)	1.21	(0.33–4.53)
Social media use × sex (girl)	0.84	(0.40–1.78)	1.85	(0.91–3.76) **	1.61	(0.75–3.44)	0.90	(0.42–1.93)

Note: Problematic social media use is included as a continuous variable, ranging from 0 to 4. The outcomes are included as binary variables, where a value of 1 indicated that the student experienced the psychosomatic complaint multiple times a week. The analysis is adjusted for covariates, i.e., household income, highest educational attainment of the parents, and having a migration background. The parameters for these covariates are not presented. * *p*-value < 0.1, ** *p*-value < 0.05, and *** *p*-value < 0.01. Results are presented as Odds Ratios (ORs), including the 99% confidence interval (CI).

**Table A7:** Potential moderators of the association between problematic social media use and psychosomatic complaints (N = 585).

	General Life Satisfaction	
	B	99% CI
Model 1		
Intercept	9.05	(8.71; 9.40) ***
Problematic social media use	−0.65	(−0.85; −0.44) ***
Model 2		
Intercept	9.07	(8.73; 9.40) ***
Problematic social media use	−0.54	(−0.74; −0.33) ***
Family support (low)	−2.04	(−3.08; −1.01) ***
Social media use × family support (low)	0.15	(−0.43; 0.72)
Model 3		
Intercept	9.18	(8.79; 9.57) ***
Problematic social media use	−0.53	(−0.77; −0.29) ***
Support from friends (low)	−0.45	(−1.12; 0.23) *
Social media use × support from friends (low)	−0.17	(−0.60; 0.25)
Model 4: Teacher support		
Intercept	9.15	(8.79; 9.51) ***
Problematic social media use	−0.62	(−0.85; −0.40) ***
Teacher support (low)	−1.05	(−2.10; −0.01) **
Social media use × teacher support (low)	0.21	(−0.34; 0.76)
Model 5		
Intercept	9.05	(8.68; 9.41) ***
Problematic social media use	−0.51	(−0.76; −0.25) ***
Support from classmates (low)	−0.82	(−1.57; −0.08) ***
Social media use × support from classmates (low)	−0.17	(−0.61; 0.26)
Model 6		
Intercept	8.95	(8.49; 9.41) ***
Problematic social media use	−0.57	(−0.88; −0.25) ***
Sex (girl)	−0.13	(−0.76; 0.50)
Social media use × sex (girl)	−0.15	(−0.56; 0.27)

Note: Problematic social media use is included as a continuous variable, ranging from 0 to 4. The analysis is adjusted for covariates, i.e., sex, household income, highest educational attainment of the parents, and having a migration background. The parameters for these covariates are not presented. * *p*-value < 0.1, ** *p*-value < 0.05, and *** *p*-value < 0.01. Results include the 99% confidence interval (CI).
